# High percentage of smudge cells in a patient with COVID19: Rediscovering their utility

**DOI:** 10.1002/jha2.52

**Published:** 2020-07-03

**Authors:** Joaquín Jerez, Daniel M. Ernst

**Affiliations:** ^1^ Internal Medicine department School of Medicine Pontificia Universidad Católica de Chile Santiago Chile; ^2^ Hematology and Oncology department School of Medicine Pontificia Universidad Católica de Chile Santiago Chile; ^3^ Institute for Biological and Medical Engineering Schools of Engineering Biological Sciences and Medicine Pontificia Universidad Católica de Chile Santiago Chile

**Keywords:** biological aspects, blood morphology, chronic lymphocytic leukemia

## Abstract

We present a patient with SARS‐CoV‐2 infection, with an unexpected presence of lymphocytosis. Examination of blood film revealed mature small lymphocytes associated with high percentage of smudge cells (63%). A peripheral flow cyometry evidenced a CD5 negative CLL. A high percentage of smudge cells is associated with CLL diagnosis and has an important prognostic value: better survival and prolonged time to first treatment. It is a useful index in developing countries with low access to molecular testing.

A 55‐year‐old man with a previous history of hypertension presented with myalgia, fever, dyspnea, and mild hypoxemia. A chest X‐ray evidenced a typical pattern of COVID‐19, and a positive PCR confirmed SARS‐CoV‐2 infection. Laboratory workup showed mild leukocytosis (14 000/mm^3^) with absolute lymphocytosis (56%; 7880/mm^3^), thrombocytosis (507 000/mm^3^), and normal erythrocytes (hemoglobin, 14.4 g/dL). During hospitalization lymphocytosis diminished to 6390/mm^3^, but persisted. In this scenario, a direct visualization of blood film was performed and small mature lymphocytes with scant cytoplasm were noted (Figure 1A,B [Digital microscopy; Cellavision, ×100 objective]), with a high percentage of smudge cells (representing 63% of total lymphocyte; Figure 1C,D). A peripheral blood flow cytometry was performed (panel B [BD FACS Canto II]) that was positive for a clonal B‐cell lymphocytosis. The population of interest (Figure 2, in red) was positive for CD20^(dim)^, CD23^(high)^, CD200^(high)^, and CD43^(dim)^, and was negative for CD5, CD79b, and cell‐surface light chain expression. A diagnosis of CD5‐negative early‐stage CLL was made. Total immunoglobulins were in normal range and after 7 days of hospital care, the patient continued to recover.

**FIGURE 1:**
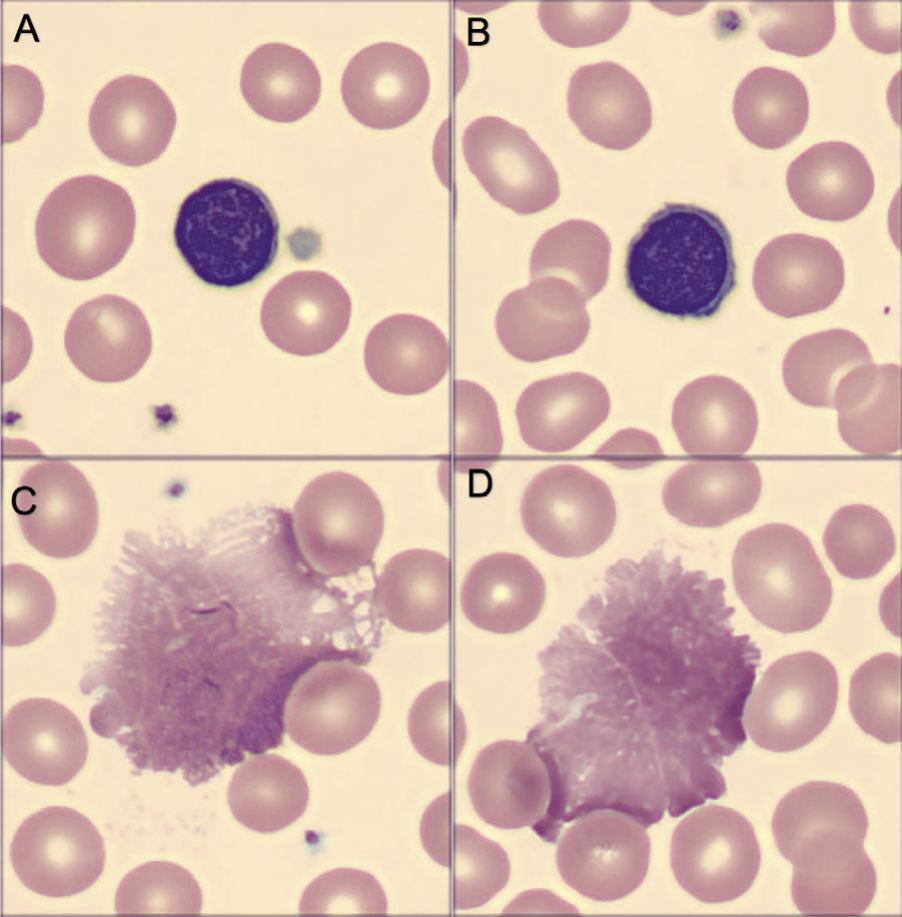
Peripheral Blood Film; digital microscopy; Cellavision, ×100 objective. A‐B, mature lymphocytes. C‐D, smudge cells.

**FIGURE 2:**
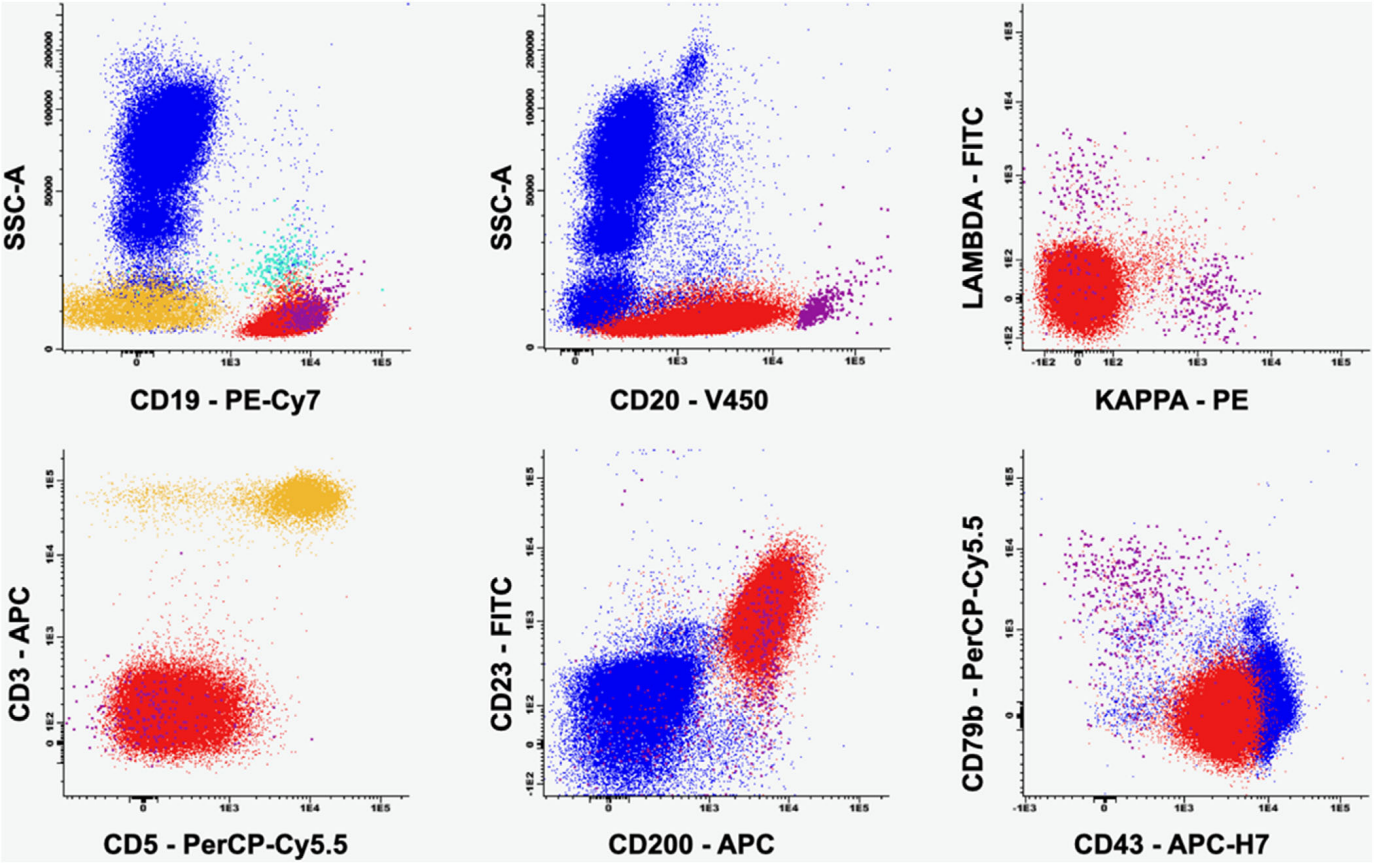
Peripheral Blood Flow Cytometry; BD FACS Canto II. Red, monoclonal B cells. Violet, normal residual B cells. Yellow, normal T cells. Blue, granulocytes and monocytes. Light‐blue, normal plasma cells.

Diagnosis of CLL is suspected in patients with absolute lymphocytosis, which contrasts the characteristic pattern of lymphopenia in COVID‐19. In association with characteristic mature small lymphocytes, an elevated percentage of smudge cells (> 30%) suggested CLL with moderate specificity [1], and correlates with better prognosis and prolonged time to first treatment [2, 3, 4]. Interestingly, the characteristic ease with which these cells are broken, more than a laboratory artefact is related with the amount of vimentin cellular content [4]. In developing countries with low access to molecular testing, clinical elements such as percentage of smudge cells, palpable adenopathies and absolute lymphocytosis over 15 000/mm^3^ are useful to stratify the risk of progression in early CLL [5].
